# Regeneration and Repair in Endodontics—A Special Issue of the Regenerative Endodontics—A New Era in Clinical Endodontics

**DOI:** 10.3390/dj4010003

**Published:** 2016-02-27

**Authors:** Tarek Mohamed A. Saoud, Domenico Ricucci, Louis M. Lin, Peter Gaengler

**Affiliations:** 1 Department of Conservative Dentistry and Endodontics, Faculty of Dentistry, University of Benghazi, El Salmania, Abn Alathera Street No. 113, Benghazi 00218, Libya; tarek_saoud@yahoo.com; 2 Private practice, Piazza Calvario 7, 87022 Cetraro, Italy; dricucci@libero.it; 3 Department of Endodontics, College of Dentistry, New York University, 345 East 24th Street, New York, NY 10010, USA; 4 Department für Zahn-, Mund- und Kieferheikunde, Fakultät für Gesundheit, Universität Witten/Herdecke, Alfred-Herrhausen-Strabe 50, 58448 Witten, Germany; peter.gaengler@uni-wh.de

**Keywords:** apical periodontitis, clinical symptom/sign, immature teeth, immunity, innervation, mature teeth, necrotic pulp, periapical healing, pulp tissue regeneration, regenerative endodontics, vital tissue

## Abstract

Caries is the most common cause of pulp-periapical disease. When the pulp tissue involved in caries becomes irreversibly inflamed and progresses to necrosis, the treatment option is root canal therapy because the infected or non-infected necrotic pulp tissue in the root canal system is not accessible to the host's innate and adaptive immune defense mechanisms and antimicrobial agents. Therefore, the infected or non-infected necrotic pulp tissue must be removed from the canal space by pulpectomy. As our knowledge in pulp biology advances, the concept of treatment of pulpal and periapical disease also changes. Endodontists have been looking for biologically based treatment procedures, which could promote regeneration or repair of the dentin-pulp complex destroyed by infection or trauma for several decades. After a long, extensive search in *in vitro* laboratory and *in vivo* preclinical animal experiments, the dental stem cells capable of regenerating the dentin-pulp complex were discovered. Consequently, the biological concept of ‘regenerative endodontics’ emerged and has highlighted the paradigm shift in the treatment of immature permanent teeth with necrotic pulps in clinical endodontics. Regenerative endodontics is defined as biologically based procedures designed to physiologically replace damaged tooth structures, including dentin and root structures, as well as the pulp-dentin complex. According to the American Association of Endodontists’ Clinical Considerations for a Regenerative Procedure, the primary goal of the regenerative procedure is the elimination of clinical symptoms and the resolution of apical periodontitis. Thickening of canal walls and continued root maturation is the secondary goal. Therefore, the primary goal of regenerative endodontics and traditional non-surgical root canal therapy is the same. The difference between non-surgical root canal therapy and regenerative endodontic therapy is that the disinfected root canals in the former therapy are filled with biocompatible foreign materials and the root canals in the latter therapy are filled with the host's own vital tissue. The purpose of this article is to review the potential of using regenerative endodontic therapy for human immature and mature permanent teeth with necrotic pulps and/or apical periodontitis, teeth with persistent apical periodontitis after root canal therapy, traumatized teeth with external inflammatory root resorption, and avulsed teeth in terms of elimination of clinical symptoms and resolution of apical periodontitis.

## 1. Introduction

Caries is the most common cause of pulp-periapical disease. When the pulp tissue involved in caries becomes irreversibly inflamed and progresses to necrotic, the only treatment option is root canal therapy because the infected necrotic pulp in the root canal system is not accessible to the host’s innate and adaptive immune defense mechanisms and antimicrobial agents. Therefore, the infected necrotic pulp tissue must be removed from the canal space by pulpectomy to prevent development or persistence of apical periodontitis.

It was the most acceptable treatment strategy for teeth with infected or non-infected necrotic pulps that the disinfected root canal space should not be left empty and should be filled with biocompatible material to prevent reinfection of the canal space for many decades. The root canal filling was expected to prevent coronal leakage, retard bacterial penetration from the canal space into the periapical tissues, and hopefully entomb bacteria in the canal space. Unfortunately, root canal filling is not able to achieve these desirable expectations in all endodontically treated teeth [1].

As our knowledge in pulp biology advances, the concept of treatment of pulpal and periapical disease appears to change accordingly. Endodontists have been looking for biologically based treatment procedures, which could promote regeneration of the dentin-pulp complex destroyed by infection or trauma, for several decades. After a long, extensive search in *in vitro* laboratory and *in vivo* preclinical animal experiments, multipotent dental stem cells capable of differentiating into odontoblast-like cells, such as dental pulp stem cells [2], stem cells from human exfoliated deciduous teeth [3], and stem cells from apical papilla [4], were discovered. Since then, the pulp biologists have tried to take advantage of these multipotent mesenchymal stem cells to regenerate the dentin-pulp complex. Several preclinical animal studies have demonstrated that it is possible to regenerate the dentin-pulp complex using dental pulp stem cells [5,6,7,8]. These preclinical animal studies established the basic concept of application of regenerative endodontics in clinical practice.

Long before the discovery of dental pulp stem cells capable of differentiating into odontoblast-like cells and producing the dentin-pulp complex, Nygaard-Ostby [9] was the pioneer who tried to explore the potential of regenerating tissue in the partially filled canal space of endodontically treated teeth by inducing periapical bleeding in dogs and human beings. It was found that the tissue that formed in the canal spaces was not pulp-like tissue, but fibrous connective tissue and cellular cementum [10]. Subsequently, Nevins *et al*. [11,12,13] tried to induce hard tissue formation into pulpless immature teeth with open apex using collagen-calcium phosphate gel in rhesus monkeys. These early studies laid the foundation for investigation of regenerative endodontics.

Regenerative endodontics is defined as biologically based procedures designed to physiologically replace damaged tooth structure, including dentin and root structures, as well as the pulp-dentin complex [14]. According to American Association of Endodontists’ (AAE) Clinical Considerations for a Regenerative Procedure [15], the primary goal of the regenerative procedure is elimination of clinical symptoms/signs and resolution of apical periodontitis. Thickening of the canal walls and/or continued root maturation is the secondary goal. Therefore, it can be stated that the primary goal of regenerative endodontic procedures and traditional non-surgical root canal therapy is similar. The difference between regenerative endodontic therapy and non-surgical root canal therapy is that the disinfected root canal space in the former therapy is filled with the host’s own vital tissue and the canal space in the latter therapy is filled with biocompatible foreign materials.

The purpose of this article is to review the potential of using regenerative endodontic procedures for immature and mature human teeth with necrotic pulps, teeth with persistent apical periodontitis after root canal therapy, traumatized teeth with external inflammatory root resorption, horizontal root fracture, and avulsed teeth in terms of elimination of clinical symptoms/signs and resolution of apical periodontitis and arrest of root resorption.

## 2. Revascularization and Regenerative Endodontics

The term “revascularization” was used in the studies of pulpal wound healing after replantation of immature permanent teeth [16,17]. Iwaya and associates [18] were the first to coin the term “revascularization” in their endodontic treatment of an immature permanent tooth with apical periodontitis and a sinus tract. The treatment procedures included sodium hypochlorite irrigation and intra-canal antibiotic paste (ciprofloxacin, metronidazole) medication without mechanical debridement. The treatment resulted in elimination of clinical symptoms and resolution of apical periodontitis. In addition, radiographic thickening of the canal walls and continued root development were observed [18]. Therefore, it was thought that the dentin-pulp complex was regenerated, perhaps, by some vital pulp tissue remaining in the apical area of the canal, which might have survived in the tooth clinically diagnosed as having devitalized and infected pulp [18]. Continued root development was also speculated, perhaps, due to survival of the apical papilla in apical periodontitis, because stem cells from the apical papilla were shown to be capable of differentiating into odontoblasts [4] and producing root dentin. Since the report of Iwaya and associates [18], revascularization has been performed in many human immature permanent teeth with necrotic pulps and apical periodontitis [19]. The treatments also achieved similar results as those reported by Iwaya and associates [18,19].

Induction of periapical bleeding into the canal space is a necessary step in regenerative endodontic procedures of immature permanent teeth with necrotic pulps. It was suggested that blood clots in the canal space could serve as a matrix or scaffold to promote pulp tissue wound healing [20]. Subsequently, Lovelace and associates [21] showed that provoked periapical bleeding also brought mesenchymal stem cells from the periapical area into the canal space. Blood contains many platelet-derived growth factors [22,23]. Therefore, induced periapical bleeding brings fibrin scaffold, mesenchymal stem cells, and blood-derived bioactive growth factors into the canal space. In addition, growth factors embedded in the dentin matrix are also released into the canal space after demineralization of dentin with Ethylenediaminetetraacetic acid (EDTA) rinse in regenerative endodontic procedures [24]. Stem cells, growth factors and a scaffold are the essential triad of tissue engineering or tissue regeneration. Therefore, the term “regenerative endodontics” was introduced in clinical endodontics, which also includes revascularization/revitalization to describe the treatment of immature permanent teeth with necrotic pulps.

## 3. Induction of Periapical Bleeding

Besides growth factors, blood clots, and mesenchymal stem cells, humoral (complement components, immunoglobulins, chemotaxins, antibacterial peptides) and cellular (polymorphonuclear leukocytes, macrophages) components of the innate and adaptive immune defense system are also brought into the canal space during the induction of periapical bleeding. These bioactive peptides and immune cells are contained in the blood [25]. Complement components such as C3b can opsonize bacteria and immunoglobulins can coat and localize bacteria to facilitate phagocytosis by activated polymorphonuclear leukocytes and macrophages through C3b and Fc receptors on these phagocytes. In addition, mesenchymal stem cells can secrete antimicrobial peptide LL-37 [26], up-regulate genes involved in promoting phagocytosis and bacterial killing [27], and augment the antibacterial activity of immune cells and secret large amounts of IL-6, IL-8, and MIF (macrophage migration inhibitory factor) cytokines to recruit and activate polymorphonuclear leukocytes and macrophages [28]. It was also suggested that LL-37 might contribute to regeneration of the dentin-pulp complex in regenerative endodontics [29]. Therefore, induction of periapical bleeding into the canal space during regenerative endodontic therapy may enhance antimicrobial clearance in the canal space. In addition, the possibility that residual bacteria in the canal space after regenerative endodontic therapy might be killed by immune defense mechanisms of regenerated vital tissue cannot be ruled out. This rationale is supported by the high success rate of immature permanent teeth with infected pulps and apical periodontitis after regenerative endodontic therapy [19].

## 4. Root Canal Fillings

A concern in regenerative endodontic therapy for immature or mature permanent teeth with necrotic pulps is residual bacteria remaining in the canal space after root canal disinfection because bacteria may grow without a root filling. Contemporary root canal infection control protocols, including mechanical instrumentation, sodium hypochlorite irrigation, and intra-canal medication with calcium hydroxide, are not able to eliminate all bacteria in the root canal system because of its anatomic complexity [30,31]. Calcium hydroxide, the most popular intra-canal medication in root canal therapy, has its shortcomings in eliminating intra-canal bacteria, because dentin and hydroxylapatite have inhibitory effects on the anti-microbial activity of calcium hydroxide [32,33]. The triple antibiotic paste (ciprofloxacin, metronidazole and minocycline) used in regenerative endodontic therapy of immature permanent teeth with necrotic pulps may also have limitations in killing intra-canal bacteria. It has been shown that triple antibiotic paste was capable of disinfecting the infected root dentin and eliminating bacteria *in vitro* [34,35]. However, these *in vitro* studies did not exactly simulate the clinical situation in which the teeth indicated for regenerative endodontic therapy usually have had a long-standing history of infection with well-established biofilm on the canal walls and bacteria in the dentinal tubules. An *in vivo* study also showed that triple antibiotic paste was able to eliminate most but not all bacteria in artificially infected root canals in dogs [36]. Ciprofloxacin inhibits DNA gyrase synthesis, metronidazole inhibits DNA synthesis, and minocycline inhibits protein synthesis of microbes [37]. These antibiotics are effective when microbes are in an active state of replication and synthesis of cell walls, proteins, or DNA but not in a stationary state. Therefore, residual bacteria are likely to remain in the canal space of mature or immature permanent teeth with infected necrotic pulps after root canal disinfection using sodium hypochlorite irrigation and intra-canal medication with calcium hydroxide and/or triple antibiotic paste [30,31,38]. Accordingly, it is recommended that the disinfected root canal space should be filled with biocompatible filling materials. The root canal filling is expected to seal the root canal space from communicating with the periapical tissues, prevent coronal leakage, and hopefully entomb residual bacteria in the canal space after root canal therapy. The root canal filling might accomplish one or more of these expectations, but not always all three. Otherwise, teeth with apical periodontitis after non-surgical root canal therapy should be able to achieve complete periapical healing. A systematic review of the outcome of primary root canal therapy does not support this notion [1].

It is not known that how much the root canal filling contributes to the success of non-surgical root canal therapy. Sjogren and associates [39] showed clinically in human teeth with apical periodontitis that if bacteriologic cultures of the canals before root-filling were negative, the success rate of root canal therapy was 94%. In contrast, if bacteriologic cultures were positive, the success rate was 68%. In monkey models, Fabricius and associates [40] also demonstrated that when bacteria remained after endodontic treatment, 79% of root canals showed non-healed periapical lesions, compared with 28% where no bacteria was found. These studies emphasize that control of root canal infection is more important than root-filling. It has also been shown in humans and animals that root canal filling was not necessary if the root canal infection was properly controlled and coronal leakage was prevented [41,42]. The quality of the root filling was found to be less critical for the periapical healing to occur than the presence or absence of bacteria in the canal space [40]. This indicates if the bacterial load in the canal space were able to reduce to the sub-threshold level, which might be determined by negative pre-obturation bacteriologic cultures of the canals, periapical wound healing could take place [39,40]. The host’s defense and the number and virulence of microbes determine the infection/inflammation [37]. In regenerative endodontic therapy, although the canal space is not filled with biocompatible foreign materials, induction of periapical bleeding and generation of vital tissue in the canal space may kill the residual bacteria remaining in the canal space as previously mentioned. However, it must be emphasized that effective control of root canal infection is paramount to regenerative endodontic therapy and non-surgical root canal therapy.

## 5. Size of Apical Foramen

The size of the apical foramen appears to be a major concern in regenerative endodontic therapy. It was suggested that an apical foramen at least 1.1 mm in diameter was necessary for successful revascularization of the pulp tissue in re-implanted human permanent incisors [16]. Therefore, human mature permanent teeth with completely formed root apices having necrotic pulps were considered not suitable for regenerative endodontic therapy. However, a study using an animal model showed that the size of an apical foramen 0.32 mm in diameter did not prevent revascularization and ingrowth of new tissue into canals after transplantation [43].

In studying regeneration of dental-pulp-like tissue by chemotaxis-induced cell homing, the apical foramina of the human mature permanent incisors and canines were not enlarged. Dental-pulp-like tissue was formed in the entire root canal from the root apex to the pulp chamber upon delivery of growth factors into the chemo-mechanically debrided canals of the teeth implanted in mouse dorsum [8]. Complete pulp regeneration in the canals of mature teeth with closed apices was also observed after pulpectomy by transplantation of autologous pulp CD105^+^ stem cells with stromal cell–derived factor-1 implanted in dogs [7]. Furthermore, it was shown histologically that new tissues could be generated in the canals of mature teeth with necrotic pulps and apical periodontitis after regenerative endodontic therapy when the canals were instrumented to #60 K-file in an animal model [44].

In human regenerative endodontic therapy studies of mature permanent teeth with necrotic pulps and apical periodontitis, Shah and associates [45] enlarged the apical foramen to #30 K-file, Paryani and Kim to #60 K-file [46], and Saoud and associates to #35 K-file [47]. Based on these animal and human studies, it is concluded that the size of the apical foramen does not have to be 1 mm in diameter for new tissue to grow into the canal space after regenerative endodontic therapy. The average size of cells in the human body ranges from 10–100 microns, which is much smaller than the average size of the apical foramen of human teeth (0.2–0.3 mm) and the tip diameter of a hand stainless steel ISO #10 K-file (0.10). Therefore, cells from the periapical tissues should be able to migrate into the canal space through the apical foramen. However, enlargement of the apical foramen to a large size may facilitate the ingrowth of new tissue into the canal space from the periapical tissues after regenerative endodontic therapy of mature permanent teeth.

## 6. Regenerative Endodontics and Pulp Tissue Regeneration/Replacement

**Figure 1:**
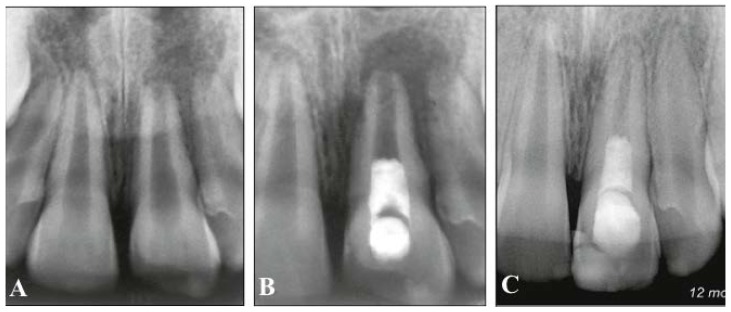
Radiographs of revascularized human immature permanent tooth #9. (**A**) Preoperative radiograph to show inflammatory periapical lesion; (**B**) Postoperative radiograph after regenerative endodontic procedures; (**C**) At 12-month follow-up, thickening of the canal walls and continued root maturation [54].

**Figure 2:**
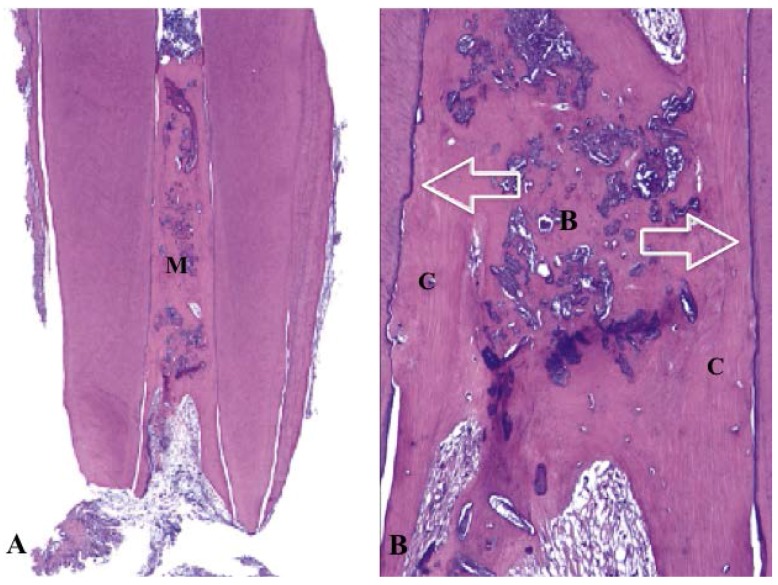
Histology of revascularized human immature permanent tooth (hematoxylin-eosin stain). (**A**) The canal space filled with mineralized tissue (M) (original magnification ×16); (**B**) High magnification of A. The mineralized tissue similar to bone (**B**) and cementum (**C**). The canal dentin walls covered by newly formed cellular cementum-like tissue (arrows) (original magnification ×100) [53].

As previously mentioned, mesenchymal stem cells in the apical papilla of immature permanent teeth with necrotic pulps introduced into the canal space during regenerative endodontic procedures might be able to differentiate into odontoblasts and produce dentin [4,21], and Hertwig’s epithelial root sheath, if intact after apical periodontitis, is capable of signaling mesenchymal stem cells in the dental follicle to differentiate into cementoblasts and regulate the root development [48,49]. Based on these presumptions, it was speculated that regenerative endodontic therapy of immature permanent teeth with necrotic pulps was able to regenerate the dentin-pulp complex and promote continued root development (Figure 1). However, histological studies of immature permanent teeth with necrotic pulps and apical periodontitis after regenerative endodontic therapy revealed that the tissues generated in the canal space were cementum-like, bone-like, or periodontal ligament–like tissue and not true pulp tissue in many animal models and humans [50,51,52,53,54,55,56] (Figure 2). Thickening of the canal walls and/or continued root maturation were due to deposition of cementum-like tissue or bone-like tissue on the canal walls and at the root apex, respectively. In one human study, nerve fibers were demonstrated in newly formed tissue in the canal space of a revascularized immature permanent tooth using immunohistochemical study [56]. Most vital tissues are supplied with blood vessels and nerve innervation because the biological function of blood vessels is largely controlled by the sympathetic and parasympathetic nervous system. Although the pulp replacement tissues are not true pulp tissue, they are vital tissues inherited with innate and adaptive immune defense mechanisms and innervated by sensory nerve fibers to detect and protect themselves from foreign invaders such as bacteria. If the primary goal of regenerative endodontic therapy of immature permanent teeth with necrotic pulps is to eliminate clinical symptoms/signs and resolve apical periodontitis, then repair by tissue different from pulp tissue, although not ideal in wound healing, is not a clinical treatment failure.

## 7. Regenerative Endodontic ProceduresSuggested by AAE [15]

First appointment:

- Local anesthesia, dental dam isolation and access.
- Copious, gentle irrigation with 20 mL NaOCl using an irrigation system that minimizes the possibility of extrusion of irrigants into the periapical space (e.g., needle with closed end and side-vent, or EndoVac™). Lower concentrations of NaOCl are advised (1.5% NaOCl (20 mL/canal, 5 min) and then irrigated with saline (20 mL/canal, 5 min), with irrigation needle positioned about 1 mm from root end, to minimize cytotoxicity to stem cells in the apical tissues.
- Dry canals with paper points.
- Place calcium hydroxide or low concentration of triple antibiotic paste. If the triple antibiotic paste is used: (1) consider sealing pulp chamber with a dentin bonding agent (to minimize risk of staining) and (2) mix 1:1:1 ciprofloxacin:metronidazole:minocycline to a final concentration of 0.1 mg/mL.
- Deliver into canal system via syringe.
- If triple antibiotic is used, ensure that it remains below Cement-enamel junction (CEJ) (minimize crown staining).
- Seal with 3–4 mm of a temporary material such as Cavit^TM^, IRM^TM^, glass-ionomer or another temporary material. Dismiss patient for one to four weeks.

Second appointment (one to four weeks after first visit):

- Assess response to initial treatment. If there are signs/symptoms of persistent infection, consider additional treatment with antimicrobial or alternative antimicrobial.
- Anesthesia with 3% mepivacaine without vasoconstrictor, dental dam isolation.
- Copious, gentle irrigation with 20 mL of 17% EDTA.
- Dry with paper points.
- Create bleeding into canal system by over-instrumenting (endo file, endo explorer) (induce by rotating a pre-curved K-file at 2 mm past the apical foramen with the goal of having the entire canal filled with blood to the level of cement-enamel junction).
- Stop bleeding at a level that allows for 3–4 mm of restorative material.
- Place a resorbable matrix such as CollaPlug™, Collacote™, CollaTape™ or other material over the blood clot if necessary and white MTA/CaOH as capping material.
- A 3–4 mm layer of glass ionomer (e.g., Fuji IlLC™, GC America, Alsip, IL, USA) is flowed gently over the capping material and light-cured for 40 s. MTA has been associated with discoloration. Alternatives to MTA should be considered in teeth where there is an esthetic concern.

* Anterior and premolar teeth—Consider use of Collatape/Collaplug and restoration with 3 mm of Resin modified glass-ionomer (RMGI) followed by bonding a filled composite to the beveled enamel margin.

* Molar teeth or teeth with Porcelain fused to metal (PFM) crown—Consider use of Collatape/Collaplug and restoration with 3 mm of MTA, followed by RMGI or alloy.

Follow-up:

Clinical and radiographic examination

* No pain, soft tissue swelling or sinus tract (often observed between first and second appointments).
* Resolution of apical radiolucency (often observed six to 12 months after treatment).
* Increased width of root walls (this is generally observed before apparent increase in root length and often occurs 12–24 months after treatment).
* Increased root length.
* Pulp vitality test.

## 8. Treatment of Immature Permanent Teeth with Necrotic Pulps

Traditionally, immature permanent teeth with necrotic pulps are treated with calcium hydroxide apexification to induce an apical hard tissue barrier formation, or with a mineral trioxide aggregate (MTA) apical plug to create a barrier [57]. The disinfected root canal space is then filled with biocompatible materials, gutta-percha and sealer/cement to the apical barrier. The outcome of calcium hydroxide or MTA apexification treatment is predictable [57]. However, the possibility of the thickening of the canal walls and/or continued root development cannot occur after apexification, thus rendering the immature teeth with already thin canal walls more prone to cervical root fracture [58,59]. In addition, calcium hydroxide apexification usually takes several months to complete, which creates a problem for children and their parents to comply with treatment procedures.

Since the introduction of regenerative endodontic therapy for immature permanent teeth with apical periodontitis by Iwaya and associates [18], regenerative endodontic therapy has become a treatment option for immature permanent teeth with necrotic pulps [19]. The treatment could result in resolution of apical periodontitis and elimination of clinical symptoms, and in some cases radiographic thickening of the canal walls and/or continued root development [19]. Thickening of the canal walls and/or increased root length are considered to be the favorable outcomes of regenerative endodontic therapy of immature permanent teeth with necrotic pulps, because they may strengthen the root and increase the root/crown ratio. Although randomized, prospective studies of regenerative endodontic therapy of immature permanent teeth with necrotic pulps are still lacking, the best available evidence seems to indicate that regenerative endodontic therapy is a feasible treatment option for immature permanent teeth with necrotic pulps [60]. Importantly, the vitality, immunity, and sensibility of immature permanent teeth with necrotic pulps are restored after regenerative endodontic therapy.

## 9. Treatment of Mature Permanent Teeth with Necrotic Pulps

Mature permanent teeth with necrotic pulps are traditionally treated with pulpectomy and root canal filling. The outcome of primary root canal treatment varies considerably depending on the presence or absence of apical periodontitis [1]. The major contributing factor to the success of root canal treatment is effective control of root canal infection [39,40]. Endodontists have enjoyed the success of practicing non-surgical root canal therapy for teeth with infected, necrotic pulps and/or apical periodontitis for many decades, given that the treatment is properly performed [39,40]. Although the technology, devices, and materials used in root canal therapy have been greatly improved, the outcome of non-surgical root canal treatment for mature permanent teeth with apical periodontitis has not been improved significantly for the past two decades [1]. This further emphasizes the importance of infection control in root canal therapy.

**Figure 3:**
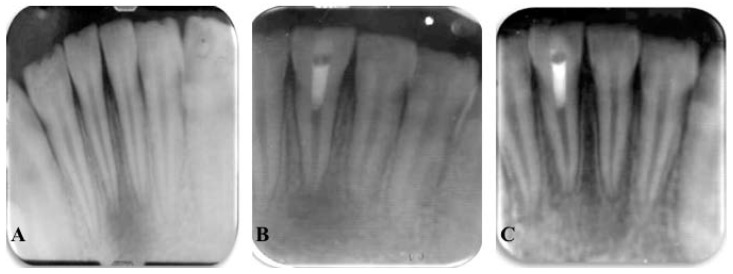
Radiographs of revascularized human mature teeth #25. (**A**) Preoperative radiograph to show inflammatory periapical lesion; (**B**) Postoperative radiograph after regenerative endodontic procedures; (**C**) At 12-month follow-up, resolution of apical periodontitis [61].

Very recently, regenerative endodontic therapy has been employed to treat mature permanent teeth with necrotic pulps and apical periodontitis, based on the rationale of elimination of clinical symptoms/signs and resolution of apical periodontitis observed in immature permanent teeth with necrotic pulps after regenerative endodontic therapy [45,46,47,61]. Thickening of the canal walls and/or continued root development are not expected to occur in mature permanent teeth following regenerative endodontic therapy. However, apical closure can take place [47]. The major difference in regenerative endodontic procedures for mature teeth with infected, necrotic pulps is that complete mechanical debridement is required to help eliminate root canal infection and remove necrotic tissue. Similar to traditional non-surgical root canal therapy, regenerative endodontic therapy of mature permanent teeth with apical periodontitis is able to result in the elimination of clinical symptoms and resolution of apical periodontitis [45,46,47,61]. Therefore, regenerative endodontic therapy provides another treatment option for mature permanent teeth with necrotic pulps (Figure 3).

## 10. Treatment of Teeth with Persistent Apical Periodontitis after Root Canal Therapy

It is well established that persistent apical periodontitis after root canal therapy is caused by persistence of root canal infection or re-infection [62,63]. Traditionally, teeth with persistent apical periodontitis after root canal therapy are managed with non-surgical root canal therapy. Endodontic surgery is usually indicated if non-surgical treatment is not feasible. The outcome of secondary root canal treatment is less favorable than that of primary root canal treatment because of several possibly complicated factors, such as untreated extra canals, ledge formation, canal blockage, separated instrument, or irretrievable cement or post in the canals created by primary root canal therapy [1,64]. In principle, secondary and primary root canal treatments are similar, except that more complete root canal infection control procedures are required in secondary root canal therapy. Recently, regenerative endodontic therapy has also been used to manage teeth with persistent apical periodontitis after root canal therapy [65,66]. The treatment also achieved elimination of clinical symptoms and resolution of apical periodontitis. Interestingly, thickening of the canal walls and apical closure were demonstrated after regenerative endodontic therapy of teeth with persistent apical periodontitis after root canal treatment [65,66]. Therefore, regenerative endodontic therapy offers another potential for retreatment of teeth with persistent apical periodontitis after root canal treatment.

## 11. Treatment of Traumatized Teeth with External Inflammatory Root Resorption, Horizontal Root Fracture, and Avulsed Teeth

Traumatized teeth with external inflammatory root resorption, horizontal root fracture, and complete avulsion are traditionally managed with control of root canal infection using chemo-mechanical debridement and root canal filling [67]. Recently, regenerative endodontic procedures have been employed to manage traumatized teeth with horizontal root fracture, external inflammatory root resorption, and complete avulsion.

In order for external inflammatory root resorption to take place, the protective layer of precementum must be damaged, likely by trauma or inflammation, thus leading to exposure of the underlying dentin [68,69,70]. In addition, the canal space has to contain infected necrotic pulp tissue. The toxic products from bacteria and tissue breakdown in the canal space diffuse through the dentinal tubules, communicating with the root surface denuded of cementum, and initiate the inflammatory reaction [68,69,70]. Therefore, infected necrotic pulp is the primary cause of external inflammatory root resorption. Treatment of external inflammatory root resorption is usually carried out by complete chemomechanical debridement, long-term calcium hydroxide dressing, and root canal filling [71]. It was presumed that calcium hydroxide would penetrate through the dentinal tubules and change the acidic environment of the resorbed root surface to prevent osteoclast activity [72,73]. It has also been shown that long-term calcium hydroxide dressing in the canal space of immature permanent teeth weakens the fragile thin root structure, thus increasing the likelihood of root fractures [59].

External inflammatory root resorption of immature permanent teeth caused by trauma was successfully treated with regenerative endodontic procedures and resulted in the resolution of apical periodontitis and the arrest of external root resorption [74]. The traumatized tooth with horizontal root fracture resulting in pulp necrosis was also successfully treated with the concept of regenerative endodontic procedures, achieving healing of the root fracture by hard tissue formation [75].

Avulsed teeth are commonly treated with immediate replantation, followed by chemomechanical debridement, calcium hydroxide dressing and root canal filling if the pulps become necrotic. Recently, an avulsed permanent mature incisor with more than 8 h extra-oral dry time was replanted into the alveolar socket after complete chemomechanical debridement of the canal space and enlargement of the apical foramen to 1.5–2 mm. The tooth was then treated with regenerative endodontic therapy, using platelet-rich plasma instead of blood clot as a scaffold. At 12-month follow-up, the tooth showed resolution of apical periodontitis and arrest of internal and external inflammatory resorption [76]. Therefore, regenerative endodontic procedures have the potential to be used to manage external inflammatory root resorption, horizontal root fracture, and avulsed tooth and should be explored further.

## 12. Treatment Outcomes of Regenerative Endodontics

**Figure 4:**
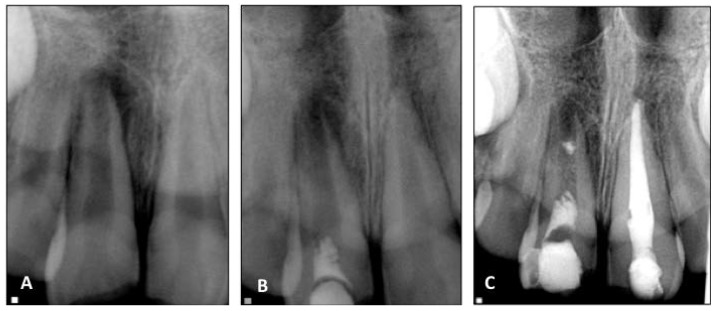
Radiographs of revascularized human immature permanent tooth #8. (**A**) Preoperative radiograph to show inflammatory periapical lesion; (**B**) Postoperative radiograph after regenerative endodontic procedures; (**C**) At 12-month follow-up, no thickening of the canal walls and no continued root maturation are seen.

There are no randomized, prospective clinical trials of regenerative endodontics for immature and mature teeth with necrotic pulps available. The level of evidence of success rates and treatment outcomes of regenerative endodontics is very low because most studies are case reports and case series [60]. In addition, there was a lack of criteria of success used in various case reports and case series of regenerative endodontic therapy. One oft-cited cohort study investigated 20 cases of regenerative endodontic therapy for immature permanent teeth with necrotic pulps and showed that the success or survival rate of treated teeth was 100% in terms of regression of clinical symptoms/signs and resolution of apical periodontitis or retention of teeth [77]. The frequency of thickening of the canal walls and/or continued root development of immature permanent teeth with necrotic pulps after regenerative endodontic therapy is not always unpredictable [78,79] (Figure 4). For mature teeth with necrotic pulps, it was demonstrated that regenerative endodontic therapy could be an alternate treatment choice to non-surgical root canal therapy regarding elimination of clinical symptoms/signs and resolution of apical periodontitis [61]. Nevertheless, long-term follow-up studies of regenerative endodontic therapy of immature and mature teeth with necrotic pulps are necessary. In addition, randomized, prospective clinical trials have to be performed to obtain reliable success rates and treatment outcomes of regenerative endodontics.

## 13. Is Regenerative Endodontic Therapy for All Teeth with Necrotic Pulps?

If the primary goal of regenerative endodontics is to eliminate clinical symptoms/signs and achieve resolution of apical periodontitis [15], then regenerative endodontic procedures can be employed to manage most teeth with necrotic pulps. Similar to non-surgical root canal therapy, if root canal infection can be effectively controlled, regenerative endodontic therapy could also be successfully performed for the immature permanent tooth with apical periodontitis in one visit [80]. However, in some teeth, regenerative endodontics may not be suitable, for example in teeth requiring a post for adequate coronal restoration. Retreatment of failure of teeth treated with regenerative endodontic procedures can be a challenge. The teeth treated with root canal filling may have a poorer survival rate than the teeth treated with regenerative procedures because of lack of defense mechanisms such as immuno-inflammatory and sensory response.

## 14. Conclusions

The goal of treatment of a disease is to assist the host’s natural wound healing processes by enhancing innate and adaptive immune defense mechanisms to eliminate irritants and create a favorable microenvironment conducive for tissue repair and/or regeneration to take place.

Infection is the main cause of primary and post-treatment apical periodontitis of immature and mature permanent teeth. Therefore, if infection is effectively under control, the tissue should be able to heal. Traditional root canal therapy of immature and mature permanent teeth with necrotic pulps is mechanically and materially based. The procedures involve removal of infected necrotic pulp, root canal disinfection, and filling of the canal space with biocompatible foreign material. Regenerative endodontic therapy is biologically based and intended to promote the host’s natural wound healing process to restore vitality, immunity, and sensitivity of tissue in the canal space destroyed by infection or trauma. Similar to traditional root canal therapy, regenerative endodontic therapy of immature and mature teeth with necrotic pulps and apical periodontitis has been shown to be able to eliminate clinical symptoms and resolve apical periodontitis. Furthermore, teeth with persistent apical periodontitis after root canal therapy can also be treated with regenerative endodontic therapy. Biologically, it may be preferable to have the disinfected root canal space filled with the host’s own vital tissues rather than with non-vital foreign materials. Like the revascularization of an immature tooth with an apical lesion reported in 2001 [18], regenerative endodontics for mature permanent teeth with necrotic pulps is still in the early stage of clinical trials. Nonetheless, regenerative procedures have become an important treatment choice for immature permanent teeth with necrotic pulps even though thickening of the canal walls and/or continued root maturation are not always predictable [78,79]. However, as previously mentioned, randomized, prospective clinical trials are required to compare the clinical outcomes of regenerative endodontic therapy and non-surgical root canal therapy for immature and mature teeth with necrotic pulps. The clinicians have to constantly follow the rapid advancement of regenerative endodontics to make appropriate treatment choices for the patients. Pulp biology and clinical endodontic therapy are slowly coming together [81]. Regenerative endodontics may bring about a new era in clinical endodontics as an alternative treatment option to non-surgical root canal treatment.
